# Application of Ultrasonic Sensors in Road Surface Condition Distinction Methods

**DOI:** 10.3390/s16101678

**Published:** 2016-10-12

**Authors:** Shota Nakashima, Shingo Aramaki, Yuhki Kitazono, Shenglin Mu, Kanya Tanaka, Seiichi Serikawa

**Affiliations:** 1 Graduate School of Sciences and Technology for Innovation, Yamaguchi University, 2-16-1, Tokiwadai, Ube-city, Yamaguchi 755-8611, Japan; w001wc@yamaguchi-u.ac.jp (S.A.); ktanaka@yamaguchi-u.ac.jp (K.T.); 2 Department of Creative Engineering, National Institute of Technology, Kitakyushu College, 5-20-1, Shii, Kokuraminami-ku, Kitakyushu-city, Fukuoka 802-0985, Japan; kitazono@kct.ac.jp; 3 Department of Electronic Control Engineering, National Institution of Technology, Hiroshima College, 4272-1, Higashino, Osakikamijima-cho, Toyota-gun, Hiroshima 725-0231, Japan; mshenglin@hiroshima-cmt.ac.jp; 4 Department of Electrical and Electronic Engineering, Kyushu Institute of Technology, 1-1, Sensui-cho, Tobata-ku, Kitakyushu-city, Fukuoka 804-8550, Japan; serikawa@elcs.kyutech.ac.jp

**Keywords:** surface condition distinction, ultrasonic sensor, reflection intensity, movement support system, accidents involving falls, road surface condition, puddle condition, elderly individuals, visually impaired individuals

## Abstract

The number of accidents involving elderly individuals has been increasing with the increase of the aging population, posing increasingly serious challenges. Most accidents are caused by reduced judgment and physical abilities, which lead to severe consequences. Therefore, studies on support systems for elderly and visually impaired people to improve the safety and quality of daily life are attracting considerable attention. In this study, a road surface condition distinction method using reflection intensities obtained by an ultrasonic sensor was proposed. The proposed method was applied to movement support systems for elderly and visually impaired individuals to detect dangerous road surfaces and give an alarm. The method did not perform well in previous studies of puddle detection, because the alert provided by the method did not enable users to avoid puddles. This study extended the method proposed by previous studies with respect to puddle detection ability. The findings indicate the effectiveness of the proposed method by considering four road surface conditions. The proposed method could detect puddle conditions. The effectiveness of the proposed method was verified in all four conditions, since users could differentiate between road surface conditions and classify the conditions as either safe or dangerous.

## 1. Introduction

There has been a rapid increase in the number of elderly individuals worldwide, and the problem of an aging society has become severe in recent years. Accidents involving elderly individuals, caused by reduced judgement and physical abilities, have increased. In addition, accidents involving visually impaired individuals have increased, thus posing a social problem.

Movement support systems are necessary to improve the safety of elderly and visually impaired people in their daily lives. Real applications of movement support systems include the employment of wheelchairs and walking canes for movement support. However, individuals moving without the help of a caretaker face a high risk of accidents (such as falling), despite using movement support systems. Hence, it is necessary to improve the safety of movement support systems.

Previous studies [1,2,3,4] involved the implementation of ultrasonic sensors to support the movements of elderly and visually impaired individuals. For example, studies examined barrier detection to improve the safety of user movements [5,6]. However, these methods are unable to detect dangerous ground surfaces. Furthermore, studies also examined material distinction characteristics [7,8]. The methods examined the reflected waveform as a 2D image, and introduced a pattern-matching approach based on benchmark patterns. However, the study limitations included a narrow distance range and difficulty in dealing with phase differences. Additionally, extant research used safety light curtains to detect dangerous regions [9]. However, light curtain systems involved high-costs, given their large scale.

In order to solve the above-mentioned problems, a previous study investigated a material distinction method that used reflection intensities obtained by an ultrasonic sensor [10]. The method was developed to detect dangerous road surfaces and to raise alerts regarding the existence of these surfaces to prevent elderly individuals from falling. However, the method involved misrecognitions in puddle detection, and it was not possible for the user to avoid surfaces with puddles in the absence of accurate alerts related to puddles. Therefore, the present study extends the results obtained by previous research to propose an improved detection method that considers the puddle condition. The effectiveness of the proposed method was verified based on experimental results. The results indicated that the proposed method was a useful road surface condition distinction method that could be applied in movement support systems, and was effective in helping elderly and visually impaired individuals avoid accidents, such as falling.

The paper is organized as follows. Section 1 presents an overview of the literature and a discussion of the problem. In Section 2, the road surface condition distinction method is introduced. The proposed distinction method with respect to the puddle condition is examined in Section 3, and the effectiveness of the proposed method is evaluated based on the experimental results. Section 4 discusses the conclusions.

## 2. Road Surface Condition Distinction Method Using Reflection Intensities Obtained by Ultrasonic Sensor

### 2.1. Introduction of Ultrasonic Sensor

An ultrasonic sensor is a type of sensor used to detect acoustic energy at wide frequency ranges exceeding 20 kHz. Ultrasonic sensors can be divided into different types, according to the differences in their applications. Generally, there are two main types of ultrasonic sensors. The first type of ultrasonic sensor is an active type, which detects the reflection of ultrasonic waves sent by the sensor itself. The applications of an active type of ultrasonic sensor include range finders, fish-finders, fathometers, sonars, and medical diagnosis devices. The other type of ultrasonic sensor is passive, which only receive ultrasonic waves. They are applied in detecting leaks in gas pipes and water pipes and in detecting corona discharges owing to insulation failure. This study involved the examination of an aerial ultrasonic sensor. 

**Figure 1:**
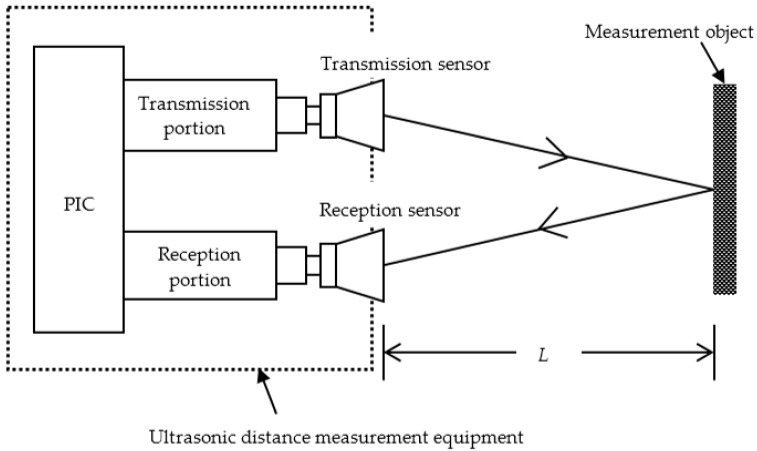
Structure of the independent type ultrasonic sensor.

**Figure 2:**
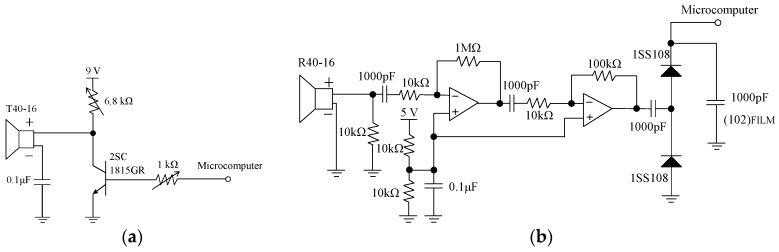
Circuit configuration for ultrasonic sensor: (**a**) Transmission portion; **(b**) Reception portion.

Range finders use ultrasonic waves [11] as distance detectors that employ reflection characteristics and the lag time between sending and receiving ultrasonic wave. Sending and receiving units of ultrasonic waves are required when an ultrasonic sensor is used to construct a distance detector. A single unit structure applies a common unit for sending and receiving; this is in contrast to an independent unit structure, which involves two separate units for sending and receiving. This study used an independent unit structure, as an ultrasonic sensor with an independent structure displays less interference and deterioration. Figure 1 shows the structure of the independent type ultrasonic sensor. Figure 2 shows the circuit configuration for the transmission portion and the reception portion of the ultrasonic sensor. The distance between the sensor and the object is estimated according to the following equations:

(1) V = 331.5+0.6 t,

(2) $$L\;=\;V\;\cdot \frac{s}{2}.$$

In the equations above, *V* (m/s) denotes the velocity of sound, *t* (°C) denotes the temperature, *S* (s) denotes the arrival time of ultrasonic wave, and *L* (m) denotes the distance between the sensor and the object. 

### 2.2. Road Surface Condition Distinction Method Using Ultrasonic Sensor

**Figure 3:**
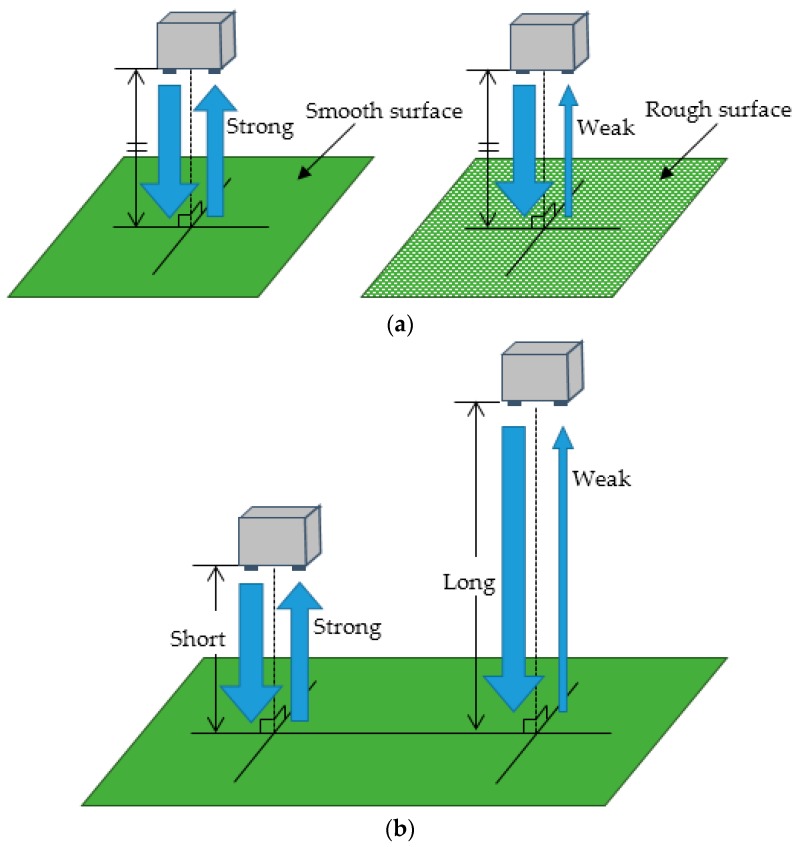
Characteristics of reflection influenced by roughness and detecting distance: (**a**) Difference in reflection intensities by surface roughness; (**b**) Difference in reflection intensities by distance detection.

The ultrasonic wave reflects disorder if roughness is present on the object surface. In this manner, the reflection of the ultrasonic wave decays. Hence, the roughness of the object can evidently affect the reflection of the ultrasonic wave. Additionally, the detecting distance influences the reflection intensity of the ultrasonic wave. Therefore, the present study proposed the application of a road surface condition distinction method that employed reflection characteristics involving relationships between object roughness and detecting distance. Figure 3 shows the characteristics of reflection influenced by the roughness and detecting distance. As shown in Figure 3, given that the reflection intensity and the detecting distance can be obtained by the ultrasonic wave reflection between the sending unit and the receiving unit, the condition of the road surface can be recognized by measuring reflection intensity and detecting distance in the proposed method.

## 3. Evaluation Experiments of the Proposed Method

### 3.1. Purpose of the Experiment

A testing experiment was implemented in the proposed method to detect puddle condition on the road. The experimental results were evaluated, and they confirmed the effectiveness of the proposed method in detecting the road conditions.

### 3.2. Experimental Methods and Conditions

**Figure 4:**
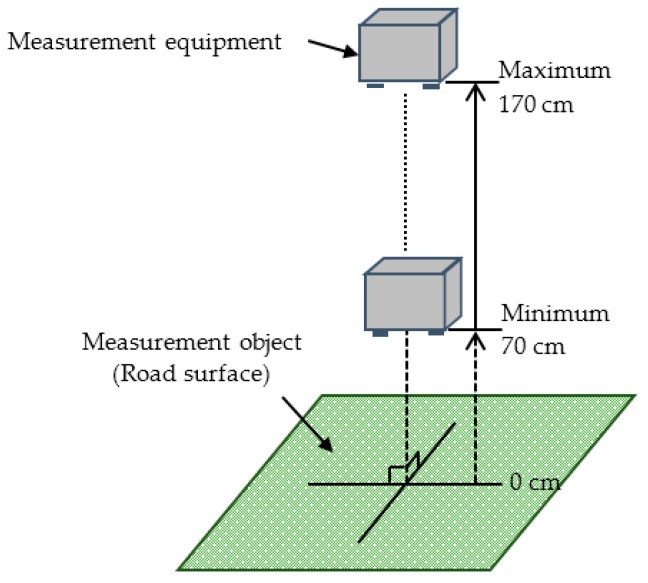
Experimental environment.

**Figure 5:**
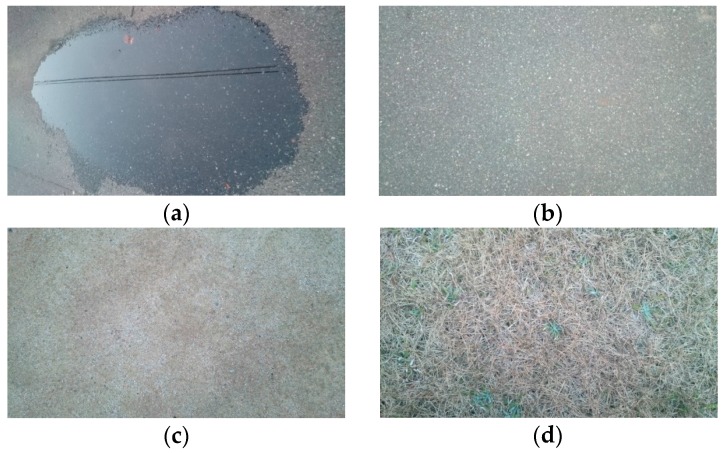
Measurement object: (**a**) Puddle; (**b**) Asphalt; (**c**) Soil; (**d**) Lawn.

An independent type T/R40-16 ultrasonic sensor (produced by NIPPON CERAMIC CO., LTD, Tottori, Japan) with a center frequency of 40 kHz (a wave length of 8.5 mm) and −6 dB directivity of 50 deg was applied as the sending and receiving unit in the detection of reflection intensity. The experimental environment is shown in Figure 4. Table 1 lists the setting of detecting distances based on the objectives. Figure 5 shows the measurement object. The experiment involved testing different types of road surface conditions, such as puddles, asphalt surfaces, soil, and lawns. The reflection intensity of each type of road surface condition was measured according to the testing distances listed in Table 1. Given that the reflection intensity was not sufficiently strong, the detecting distance for soil and lawn were set in a narrow range. Furthermore, the incident angle of the ultrasonic wave was set at 90°. The experiment was conducted on a sunny day with a temperature of 13 °C and a humidity of 57%.

### 3.3. Experimental Results and Study

**Figure 6:**
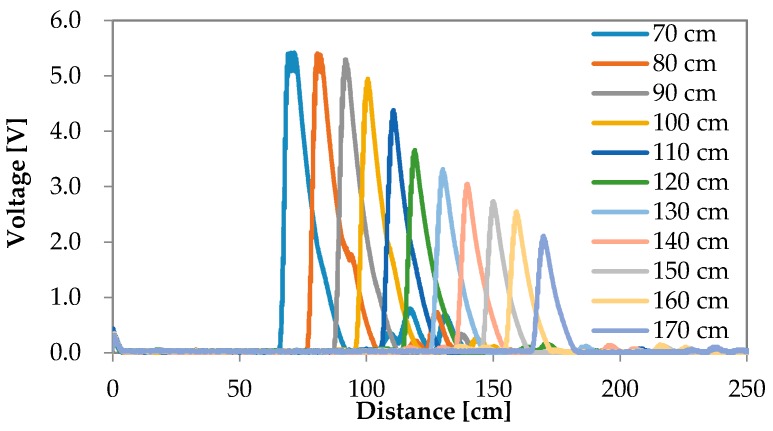
Experimental results of the reflection wave with respect to puddle.

**Figure 7:**
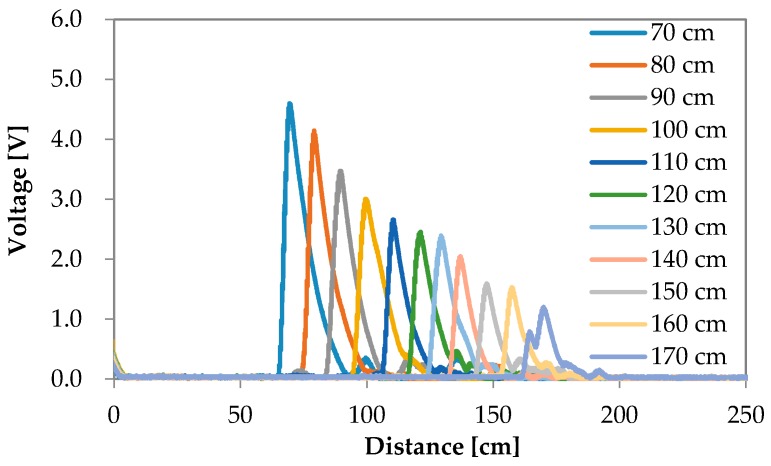
Experimental results of the reflection wave with respect to asphalt.

**Figure 8:**
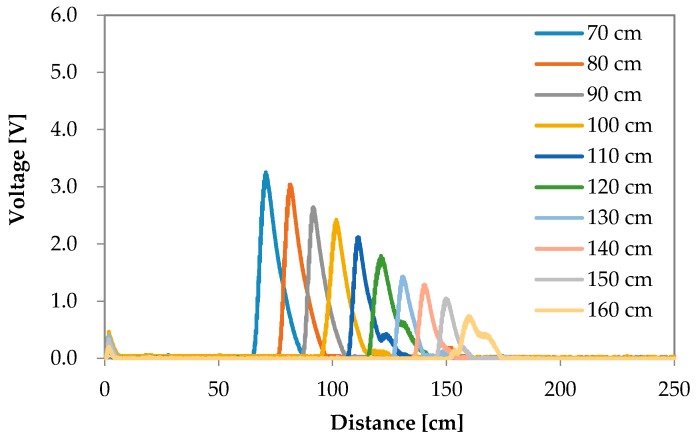
Experimental results of the reflection wave with respect to soil.

**Figure 9:**
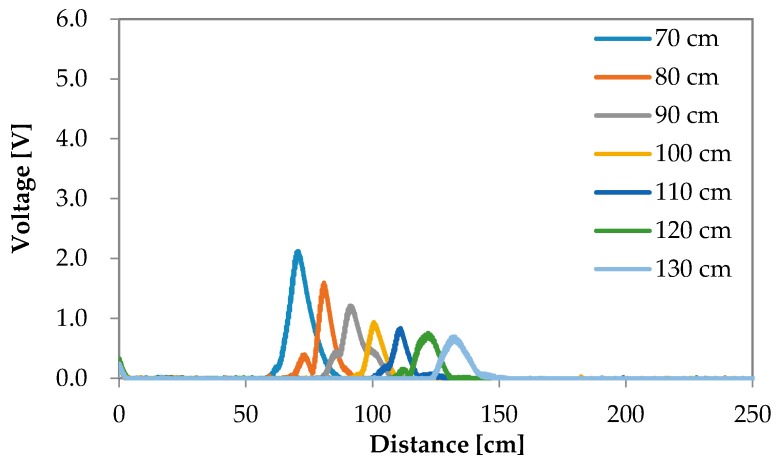
Experimental results of the reflection wave with respect to lawn.

**Figure 10:**
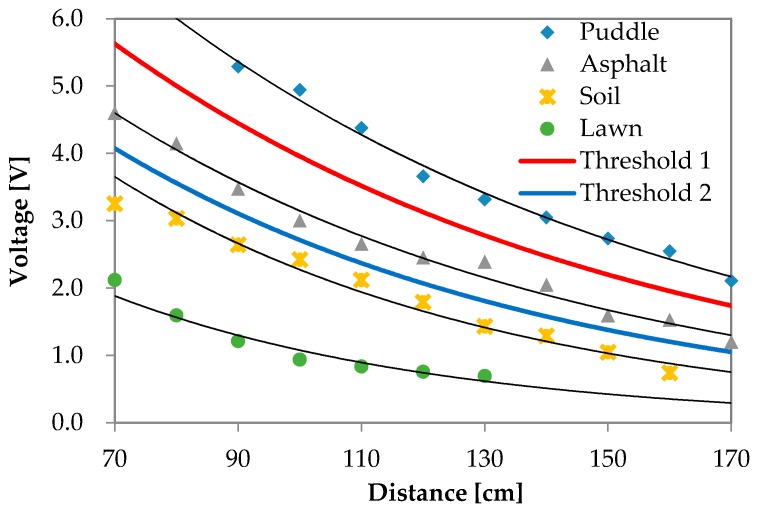
Experimental result showing the reflection intensities of all objects.

The reflection waves for each road surface condition are shown in Figure 6, Figure 7, Figure 8 and Figure 9. The waves are summarized in Figure 10.

The exponential approximations of the detected data are expressed in Equations (3)–(6). Equation (3) represents the approximation of the puddle condition, Equation (4) represents the approximation of the asphalt condition, Equation (5) represents the approximation of the soil condition, and Equation (6) represents the approximation of the lawn condition.

(3) $$v\;=\;14.837\;e^{-0.011d},$$

(4) $$v\;=\;11.138\;e^{-0.013d},$$

(5) $$v\;=\;11.045\;e^{-0.016d},$$

(6) $$v\;=\;6.9184\;e^{-0.019d}.$$

In the equations, *v* (V) denotes the reflection intensity, and *d* (cm) denotes the detecting distance. In Equation (3), since the detecting ranges of 70 cm and 80 cm for the puddle exceed the possible ranges, they were not included in the approximation.

The results shown in Figure 10 clearly indicate that the reflection intensity decayed at an exponential rate according to the increase in detecting distance. Additionally, the reflection observed in the puddle condition was most intensive when compared with that of other conditions. The experimental conditions in decreasing order of reflection intensities were ranked as asphalt, soil, and lawn. The results confirmed that the intensity of a smooth surface was high and that the intensity of a rough surface was low. The findings revealed that the characteristics of an ultrasonic wave included disordered reflection for an object involving rough surface.

Threshold 1 between asphalt and puddle could be synthesized based on the experimental results as represented by the red solid line shown in Figure 10. Threshold 2 between asphalt and soil could be synthesized as shown by the blue solid line in Figure 10. Threshold 1 is expressed by Equation (7), and Threshold 2 is expressed by Equation (8), as given below:

(7) $$v\;=\;12.797\;e^{-0.012d},$$

(8) $$v\;=\;10.518\;e^{-0.014d}.$$

Road conditions were recognized by using distinction thresholds. If the detected intensity exceeded Threshold 1 (Equation (7)), it was identified as a puddle. If the detected intensity was lower than Threshold 2 (Equation (8)), it was identified as lawn or soil. If the detected intensity was between Threshold 1 (Equation (7)) and Threshold 2 (Equation (8)), it was identified as asphalt.

The approach detailed in this study could be used to divide the recognition of a road surface according to three conditions. The safety condition was identified using the information obtained by the proposed method. Typically, asphalt ground was considered safe, while the probability of slipping and falling on a puddle was high, especially for elderly and visually impaired individuals. The users can avoid dangerous road surfaces by employing the proposed method to effectively distinguish between road surface conditions.

## 4. Conclusions 

This study involved the development of a road surface condition distinction method by considering puddle conditions. Four types of road surface conditions were investigated in this study. The effectiveness of the proposed method was verified in all four conditions. The experimental results indicated that two thresholds as defined by the proposed method could be used to identify road surface conditions. The road surface conditions could be recognized as either safe or dangerous. The effectiveness of the proposed method was confirmed by the experimental findings.

Thus, employing the proposed method in road surface condition distinction for movement support systems, such as wheelchairs and walking canes, can help prevent accidents (e.g., falling down) for elderly and visually impaired individuals. The proposed method can also to be applied to automobiles.

## Figures and Tables

**Table 1:** Setting of detecting distances based on the objectives.

Measurement Object	Range of Measurement Distance (cm)		
	Minimum	Maximum	Interval
Puddle	70	170	10
Asphalt	70	170	10
Soil	70	160	10
Lawn	70	130	10

## References

[1] Masuzawa T., Tanaka K., Minami S. (2009). Development of a safety driving system for electric wheelchair. J. Asian Electr. Veh..

[2] Mazo M., Rodriguez F.J., Lazaro J.L., Urena J., Garcia J.C., Santiso E., Revenga P., Garcia J.J. (1995). Wheelchair for physically disabled people with voice, ultrasonic and infrared sensor control. Auton. Robots.

[3] Kadono Y., Dobashi H., Abe T., Tajima T., Kimura H. A fall detection system in bathroom using ultrasound sensor. Proceedings of the 71th Annual Convention IPS Japan.

[4] Ohnishi Y., Abe T., Nambo H., Kimura H., Ogoshi Y. (2006). Development of abnormality detection system for bathers using ultrasonic sensors. IEEJ Trans. Sens. Micromach..

[5] Takegami T. (2010). Fundamental study of surrounding situation sensing for visually handicapped person using ultrasonic ranging sensors. Fac. Hum..

[6] Kitahara S., Morioka H., Niitsu Y. Obstacle detection and notification method on walking support for visually handicapped persons. Proceedings of the IEICE Student Activity Committee Workshop, Tokyo Section.

[7] Moritake Y., Hikawa H. (2002). Hardware material recognition system using combinatorial logic circuit and ultrasonic sensor. IEICE Trans..

[8] Moritake Y., Hikawa H. (2004). Category Recognition System Using Two Ultrasonic Sensors and Combinational Logic Circuit. IEICE Trans..

[9] Lu H., Serikawa S. (2013). Design of freely configurable safety light curtain using hemispherical mirrors. IEEJ Trans. Electr. Electron. Eng..

[10] Iwasaki D., Haruyama K., Mu S., Lu H., Tanaka K., Kitazono Y., Wakasa Y., Serikawa S., Nakashima S. Ground material distinction method using reflection intensities obtained by ultrasonic sensor. Proceedings of the 2012 IEEE/SICE International Symposium on System Integration (SII).

[11] Ishihara M., Shiina M., Suzuki S.-N. (2009). Evaluation of method of measuring distance between object and walls using ultrasonic sensors. J. Asian Electr. Veh..

